# 2-carba cyclic phosphatidic acid suppresses inflammation *via* regulation of microglial polarisation in the stab-wounded mouse cerebral cortex

**DOI:** 10.1038/s41598-018-27990-1

**Published:** 2018-06-26

**Authors:** Kei Hashimoto, Mari Nakashima, Ayana Hamano, Mari Gotoh, Hiroko Ikeshima-Kataoka, Kimiko Murakami-Murofushi, Yasunori Miyamoto

**Affiliations:** 1 Graduate School of Humanities and Sciences, Ochanomizu University, Ohtsuka, Bunkyo-ku, Tokyo, Japan; 2 Institute for Human Life Innovation, Ochanomizu University, Ohtsuka, Bunkyo-ku, Tokyo, Japan; 3 Research division of human welfare science, Ochanomizu University, Ohtsuka, Bunkyo-ku, Tokyo, Japan; 4 Research Fellow of Japan Society for the Promotion of Science, Kojimachi, Chiyoda-ku, Tokyo, Japan; 5 Faculty of Science and Engineering, Waseda University, Okubo, Shinjuku-ku, Tokyo, Japan; 6 Department of Pharmacology and Neuroscience, Keio University School of Medicine, Shinanomachi, Shinjuku-ku, Tokyo, Japan

## Abstract

Traumatic brain injury (TBI) is caused by physical damage to the brain and it induces blood-brain barrier (BBB) breakdown and inflammation. To diminish the sequelae of TBI, it is important to decrease haemorrhage and alleviate inflammation. In this study, we aimed to determine the effects of 2-carba-cyclic phosphatidic acid (2ccPA) on the repair mechanisms after a stab wound injury as a murine TBI model. The administration of 2ccPA suppressed serum immunoglobulin extravasation after the injury. To elucidate the effects of 2ccPA on inflammation resulting from TBI, we analysed the mRNA expression of inflammatory cytokines. We found that 2ccPA prevents a TBI-induced increase in the mRNA expression of *Il-1β*, *Il-6*, *Tnf-α* and *Tgf-β1*. In addition, 2ccPA reduces the elevation of Iba1 levels. These data suggest that 2ccPA attenuates the inflammation after a stab wound injury *via* the modulation of pro-inflammatory cytokines release from microglial cells. Therefore, we focused on the function of 2ccPA in microglial polarisation towards M1 or M2 phenotypes. The administration of 2ccPA decreased the number of M1 and increased the number of M2 type microglial cells, indicating that 2ccPA modulates the microglial polarisation and shifts them towards M2 phenotype. These data suggest that 2ccPA treatment suppresses the extent of BBB breakdown and inflammation after TBI.

## Introduction

Traumatic brain injury (TBI) occurs following physical brain damage due to a variety of causes such as road traffic accidents and falls. It affects approximately 10 million people worldwide per year. TBI severity and prognosis are associated with the impact of the primary and secondary brain injury. Primary brain injury refers to the mechanical damage including axonal loss and haemorrhage. Secondary brain injury occurs after several minutes to days of the primary brain injury. The secondary brain injury is caused by oedema and ischemia due to inflammation, cell death, and gliosis^1^. The secondary brain injury has a significant impact on the TBI outcome. To attenuate negative consequences of TBI, it is very important to alleviate the impact of the secondary brain injury.

Lysophosphatidic acid (LPA) is a lipid mediator. LPA was found to be elevated in the cerebrospinal fluid of TBI patients and of mice in a TBI model^2^. In a zebrafish model of spinal cord injury (SCI), LPA administration increased glial cell proliferation and neuronal cell death, indicating that LPA exerts proinflammatory activity and plays an inhibitory role in neuroregeneration after nerve injury^3^. It has been demonstrated that blocking LPA signalling with LPA-antibodies reduces lesion volume and diminishes tissue damage in mouse models of SCI and TBI^2,3^. LPA is generated by enzymatic hydrolysis of lysophosphatidylcholine catalysed by autotaxin (ATX)^4^, and it has been reported that ATX-mediated LPA production is induced 2–3 hours after nerve injury^5^. Therefore, decreasing LPA production and/or inhibiting its function after brain injury may diminish the secondary injury after TBI and thus attenuate the sequelae of TBI.

Cyclic phosphatidic acid (cPA) is a lipid mediator, which is also generated by ATX^6^. Although the chemical formula of cPA is similar to that of LPA, cPA has a unique structure consisting of a cyclic phosphate ring at the *sn*-2 and *sn*-3 positions of its glycerol backbone^7,8^. These features provide cPA with biological functions that are distinct from or oppose the functions of LPA^9,10^. In the nervous system, cPA exhibits neuroprotective effects. For example, *in vitro* studies revealed that cPA elicits neurotrophin-like actions^9,11^ and protects neurons from mitochondrial dysfunction-induced apoptosis^12^. In an *in vivo* study, cPA attenuated ischemia-induced delayed neuronal death in rat hippocampal CA1 regions^13^. Additionally, cPA reduced the extent of demyelination, astrogliosis, microglial activation, and motor dysfunction in a cuprizone-induced multiple sclerosis mouse model^14^. Moreover, it is known that cPA inhibits ATX activity^15^. Thus, cPA could be an endogenous inhibitor of LPA production *via* ATX inhibition. These results led us to investigate the effects of cPA on TBI. In this study, we used a metabolically stabilised derivative of cPA; 2-carba-cyclic phosphatidic acid (2ccPA) is a compound in which the phosphate oxygen is replaced by a methylene group at the *sn*-2 position^15,16^. It has been demonstrated that 2ccPA efficiently replicates numerous biological functions of cPA. For instance, 2ccPA is a much more potent inhibitor of ATX than cPA^15^. In a cuprizone-induced multiple sclerosis mouse model, 2ccPA not only exhibits beneficial effects on neuroregeneration, it exerts also more pronounced effects than cPA^14,17^.

Using as a TBI model a cerebral stab wound lesion caused by a needle, we investigated the effects of 2ccPA on the progression of the wound and found that 2ccPA reduces the severity of blood-brain barrier (BBB) breakdown in the primary brain injury phase. Furthermore, 2ccPA suppresses the expression of the pro-inflammatory cytokines interleukin-1β (IL-1β), interleukin-6 (IL-6), and tumour necrosis factor-α (TNF-α) and the anti-inflammatory cytokine transforming growth factor-β1 (TGF-β1) as well as the activation of microglial cells in the injured area. To further investigate the effects of 2ccPA in the stab wound model, we examined the function of 2ccPA in microglial polarisation towards a neurotoxic M1 or a neuroprotective M2 phenotype. We found that 2ccPA decreases the number of M1 microglial cells and increases the number of M2 microglial cells. These data demonstrate that 2ccPA is a promising therapeutic agent for primary and secondary traumatic brain injury.

## Materials and Methods

### Mice

Female ICR wild-type mice were obtained from Charles River Laboratory (Kanagawa, Japan). All animal experiments were approved by the Institutional Animal Care and Use Committee of Ochanomizu University, Japan (animal study protocols 15027, 16006, and 17006), and were performed in accordance with the guidelines established by the Ministry of Education, Science, and Culture in Japan. All mice were allowed free access to food and water in a clean environment.

### Pharmacological agents

2ccPA 18:1 was chemically synthesised as previously described^16^. 2ccPA was dissolved in phosphate buffered saline (PBS).

### Stab wound surgery

Stab wound surgery was performed using a modified version of the procedure previously described by Hashimoto *et al*.^18^. Mice (6 weeks old) were anaesthetised by a mixture of 0.75 mg/kg Domitor (Nippon Zenyaku Kogyo, Fukushima, Japan), 4 mg/kg midazolam (Sandoz, Holzkirchen, Germany), and 5 mg/kg Vetorphale (Meiji Seika Pharma, Tokyo, Japan). A 19-gauge needle was then used to penetrate the skull in the occipital region of the right hemisphere, and a 27-gauge needle was inserted through this hole along the rostrocaudal axis, and the needle was pulled out gently to induce just one stab wound to the right cerebral cortex. After the operation, the Domitor antagonist Antisedan (0.75 mg/kg; Nippon Zenyaku Kogyo) was injected intraperitoneally to facilitate recovery from anaesthesia. A sample size calculation was based on our previous study^18^.

### Postoperative treatment with 2ccPA

**Figure 1:**
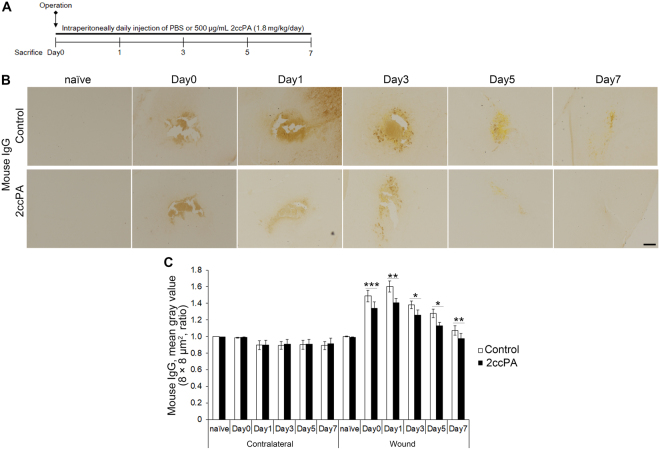
Effects of 2ccPA administration on the recovery from BBB breakdown caused by stab wound injuries. The extent of BBB breakdown near the lesion was assessed by the extravasation of serum IgG. (**A**) The dose, delivery route, and treatment regimen of 2ccPA. After the stab wound surgery, 500 µg/ml 2ccPA (1.8 mg/kg/day) or PBS as a vehicle were injected intraperitoneally. Successive injections of the same dose of 2ccPA or PBS were administered every day until the collection of the brain samples. Uninjured mice that received PBS or 2ccPA were regarded as naïve. (**B**) Cortical sections were immunoenzymatically stained with an antibody against mouse IgG. Regions near the stab wound injury are shown from naïve and stab-wounded mice treated with PBS (control) or 2ccPA. Scale bar: 100 µm. (**C**) Quantification of mouse IgG staining intensities near stab wounded injuries in PBS- and 2ccPA-treated mice on days 0, 1, 3, 5, and 7. All values were normalised to the mean staining intensity of the uninjured, contralateral hemisphere (n = 3 female mice/group). *p < 0.05, **p < 0.01, ***p < 0.001 vs. control groups, two-way ANOVA and Tukey-Kramer test. Data represent the mean ± SEM of three independent experiments using three pairs of mice at each specified day. A total of 36 female mice (6 weeks old) were subjected to this experiment.

Wounded and naïve mice were randomly assigned to receive either 2ccPA or vehicle (PBS). They received shortly after the administration of Antisedan either 500 µg/ml 2ccPA (1.8 mg/kg/day) or PBS by intraperitoneal injection. Successive injections of the same dose of PBS or 2ccPA were performed every day until the collection of the brain samples (see Fig. 1A). Naïve mice had the same drug regimen. The delivery route and dosing regimen were based on previous work and preliminary tests^14^.

### Immunohistochemistry

Immunohistochemistry was performed as described by Hashimoto *et al*. with modifications^18^. Briefly, naïve and stab-wounded mice at postoperative days 0, 1, 3, 5, and 7 were perfused with PBS and fixed with 4% paraformaldehyde (PFA), and the collected brains were fixed with 4% PFA overnight. After cryoprotection with 30% sucrose, serial coronal sections (20 μm thick) of the cerebral cortex were obtained using a cryostat (Leica CM1850; Leica Microsystems, Wetzlar, Germany).

For immunofluorescence staining of microglial cells, the sections were incubated with primary antibodies overnight followed by secondary antibodies for 1 h. The primary antibodies in this study included anti-Iba1 (1:500 dilution; 019–19741; Wako, Osaka, Japan), anti-CD86 (1:200 dilution; 14-0862-82; Thermo Fisher Scientific), and anti-CD206 (1:200 dilution; AF2535; R&D Systems, Minneapolis, MN). The secondary antibodies were as follows: Alexa Fluor 568 goat anti-rabbit immunoglobulin G (IgG), Alexa Fluor 488 goat anti-rat IgG, and Alexa Fluor 488 chicken anti-goat IgG (all Thermo Fisher Scientific). Additionally, 4′,6-diamidino-2-phenylindole dihydrochloride (DAPI; 1:2500 dilution, Roche Diagnostics) was used for nuclear staining. Images were captured with a confocal microscope (LSM710; Carl Zeiss, Oberkochen, Germany). To determine the frequency of stained cells, fields (600 × 600 μm^2^) were chosen arbitrarily near stab wound regions in each cerebral cortex section. A minimum of five sections from each mouse was analysed.

For immunoenzymatic stainings, the sections were incubated with the primary antibody overnight, with secondary antibodies for 1 h, and additionally with the avidin-biotin complex (Vectastain Elite ABC Standard Kit; Vector Laboratories, Burlingame, CA) for 1 h, followed by incubation in 3,3′-diaminobenzidine solution to detect signals. For the staining of mouse IgG, the sections were incubated with only secondary antibody for 1 h. Images of the sections were captured using a microscope (FSX100; Olympus, Tokyo, Japan). For the quantification of the mouse IgG staining, staining intensities were quantified using ImageJ by the measurement of mean grey values in five fields (8 × 8 µm^2^), which were selected randomly from immunoenzymatically stained regions in each section. To quantify the Iba1 staining intensity, staining intensities were quantified by the measurement of mean grey values in ten fields (8 × 8 µm^2^), which were selected randomly in each section. The mean grey value in each contralateral, uninjured region was subtracted from those of the ipsilateral region to correct for background values. A minimum of five sections was analysed from each mouse.

### Real-time RT-PCR

RNA was extracted from cerebral cortices near the site of the injury or cultured microglial cells. The RNA (1 μg) was reverse-transcribed using ReverTra Ace qPCR RT Kit (Toyobo Co., Ltd., Osaka, Japan). Real-time RT-PCR analysis was performed to quantify *Iba1*, *Il-1β*, *Il-6*, *Tnf-α*, *Tgf-β1*, *Lpar1–6*, *Gpr87*, and *P2ry10* mRNA expression levels. The primer sequences used in the present study are listed in Table 1^12,19–23^. mRNA expression levels were quantified using a KOD SYBR qPCR Mix (Toyobo Co., Ltd.) with an ABI 7300 real-time PCR machine (Thermo Fisher Scientific, Waltham, MA). Analyses of relative gene expression levels were performed using the 2−Delta-Delta-Ct method between contralateral and injured cortices to reduce individual differences.

**Table 1:** Primer sequences used for real-time RT-PCR.

Gene	Primer Sequence	
*Iba1*	Forward	5′-GGATTTGCAGGGAGGAAAAG-3′
	Reverse	5′-TGGGATCATCGAGGAATTG-3′
*Tnfα*	Forward	5′-ACAGAAAGCATGATCCGCG-3′
	Reverse	5′-GCCCCCCATCTTTTGGG-3′
*Il-1β*	Forward	5′-GCACACCCACCCTGCAG-3′
	Reverse	5′-AACCGCTTTTCCATCTTCTTCTT-3′
*Il-6*	Forward	5′-CTGCAAGAGACTTCCATCCAGTT-3′
	Reverse	5′-GAAGTAGGGAAGGCCGTGG-3′
*Tgf-β1*	Forward	5′-CCCTATATTTGGAGCCTGGA-3′
	Reverse	5′-CTTGCGACCCACGTAGTAGA-3′
*Cd86*	Forward	5′-GTTACTGTGGCCCTCCTCCTT-3′
	Reverse	5′-CTGATTCGGCTTCTTGTGACATA-3′
*Cd206*	Forward	5′-CCCAAGGGCTCTTCTAAAGCA-3′
	Reverse	5′-CGCCGGCACCTATCACA-3′
*Lpar*_*1*_	Forward	5′-GTCTTCTGGGCCATTTTCAA-3′
	Reverse	5′-TCATAGTCCTCTGGCGAACA-3′
*Lpar*_*2*_	Forward	5′-CCAGCCTGCTTGTCTTCCTA-3′
	Reverse	5′-GTGTCCAGCACACCACAAAT-3′
*Lpar*_*3*_	Forward	5′-GAATTGCCTCTGCAACATCTC-3′
	Reverse	5′-ATGAAGAAGGCCAGGAGGTT-3′
*Lpar*_*4*_	Forward	5′-TCTGGATCCTAGTCCTCAGTGG-3′
	Reverse	5′-CCAGACACGTTTGGAGAAGC-3′
*Lpar*_*5*_	Forward	5′-CGCCATCTTCCAGATGAAC-3′
	Reverse	5′-TAGCGGTCCACGTTGATG-3′
*Lpar*_*6*_	Forward	5′-TCTGGCAATTGTCTACCCATT-3′
	Reverse	5′-TCAAAGCAGGCTTCTGAGG-3′
*Gpr87*	Forward	5′-ACAAATCCAGCAGGCAATTC-3′
	Reverse	5′-TACGGGAGGAAGCAGGTAAA-3′
*P2ry10*	Forward	5′-TCAACATGTATGCCAGCATTT-3′
	Reverse	5′-GAAATGGCAAACAGGCAGTC-3′
*Gapdh*	Forward	5′-CGTGTTCTACCCCCAATGT-3′
	Reverse	5′-TGTCATCATACTTGGCAGGTTTCT-3′

### Microglial cell cultures

Cortical microglial cells were cultured using a modified version of the procedure described by Milner and Campbell^24,25^. Microglial cells and astrocytes were collected from the cerebral cortices of ICR mice on postnatal day 2. Cultures were maintained in T75 flasks in Dulbecco’s Modified Eagle’s Medium (Sigma-Aldrich, St. Louis, MO) with 10% fetal bovine serum, 50 units/ml penicillin and 50 μg/ml streptomycin for 10–14 days, before being shaken to yield microglial cells. Microglial cells were plated into 24-well plates (2.0 × 10^5^ cells/well) and cultured for 24 h with astrocyte-derived supernatants. Finally, 1 μg/ml lipopolysaccharide (LPS) (Sigma-Aldrich) or 1, 5 and 10 μM 2ccPA were added for 4 h. PBS was used as the vehicle for LPS.

### Statistical analyses

In the experiments using tissue sections, three pairs of 2ccPA treated mice and control mice were used. For real-time RT-PCR analysis *in vivo*, eight pairs of mice were used. The experiments using cultured microglial cells were performed a minimum of four times. The values are expressed as the mean ± standard error of the mean (SEM). Changes were considered statistically significant if the *p*-value obtained from the one-way or two-way ANOVA followed by Tukey-Kramer test or Student’s *t*-test reached one of the following significance levels: *p < 0.05, **p < 0.01, and ***p < 0.001.

## Results

### Administration of 2ccPA reduces the extent of BBB breakdown resulting from stab wounds to the mouse cerebral cortex

To elucidate whether 2ccPA affects the wound-healing process and recovery from BBB breakdown in injured regions, stab wounds were made in murine cerebral cortices using needles, and 2ccPA or PBS as a vehicle control was administered intraperitoneally immediately after the operation. Furthermore, successive injections of 2ccPA or PBS were given every 24 h until the collection of the brain samples (Fig. 1A). In the injured regions of the collected brains, the extent of BBB breakdown was assessed by measuring extravasation of serum IgG at days 0, 1, 3, 5, and 7. Serum IgG extravasation was not detected in the cortices of naïve mice but was observed around the lesions in cortices from injured mice on days 0–7 (Fig. 1B). IgG extravasation in the lesion area transiently increased after the operation until day 1 and decreased steadily afterwards from day 3 to day 7. Extravasation of serum IgG in contralateral hemispheres was not significantly induced by the stab wound injury (Fig. 1C). The administration of 2ccPA significantly decreased IgG extravasation in the lesions on days 0, 1, 3, 5, and 7 compared to control mice but did not affect extravasation in the contralateral regions (Fig. 1B,C). In addition, we profiled the mRNA expression levels of LPA receptors 1–6 (*Lpar1–6*), G protein-coupled receptor 87 (*Gpr87*), and the purinergic receptor P2Y10 (*P2ry10*), which also serve as cPA receptors, in mouse cerebral cortices after stab wound injuries. We found that the mRNA expression levels of most LPA receptors were increased and peaked around day 5 after the application of the stab wound, while the administration of 2ccPA suppressed this increase of mRNA expression (Supplementary Fig. S1).

### 2ccPA suppresses the expression of inflammatory cytokines after a traumatic injury to the cerebral cortex

**Figure 2:**
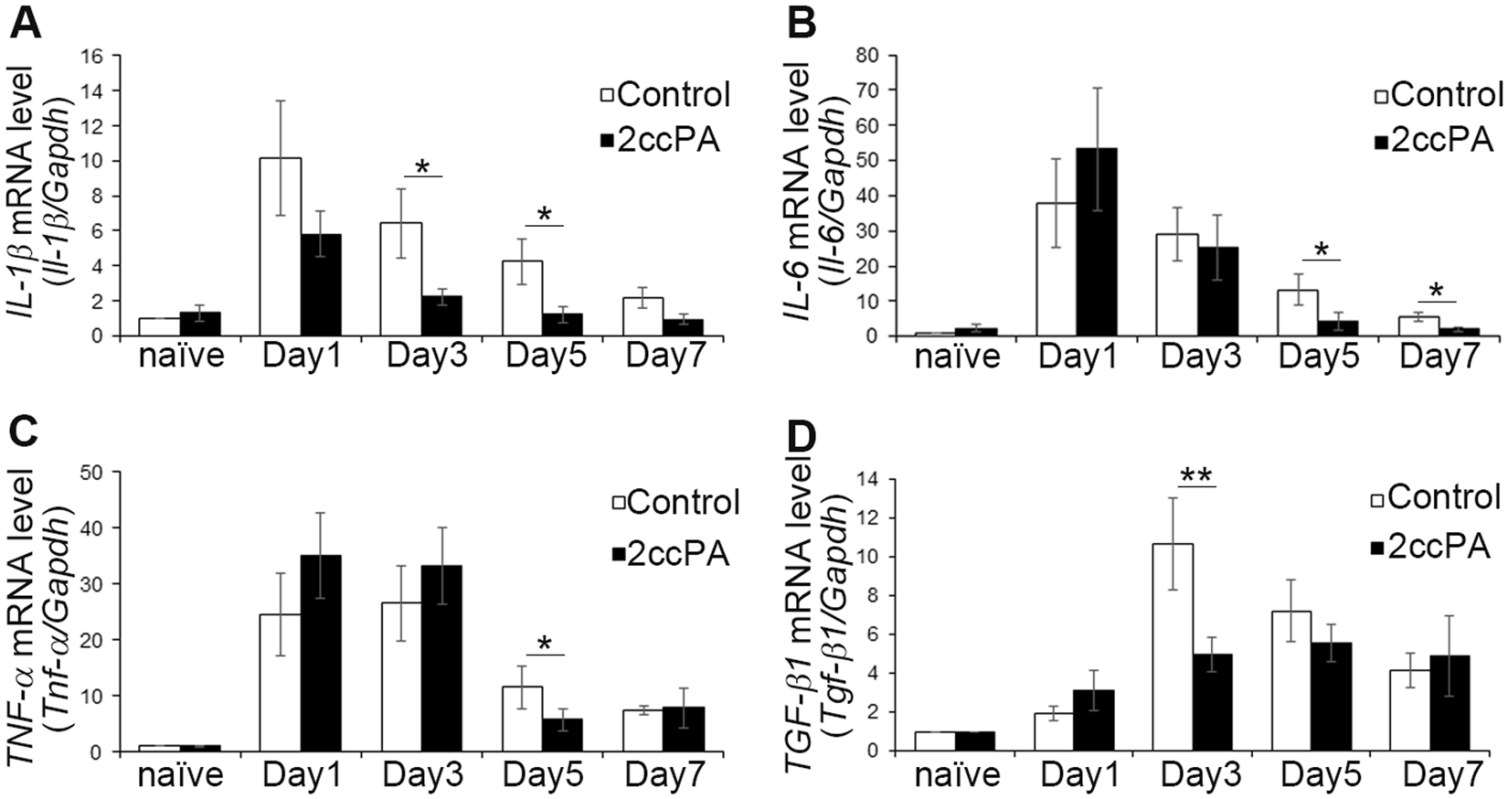
mRNA expression levels of pro- and anti-inflammatory cytokines near the stab wound injury in the cerebral cortices of 2ccPA-treated mice. (**A**–**D**) Real-time RT-PCR analyses of *Il-1β*, *Il-6*, *Tnf-α*, and *Tgf-β1* mRNA expression on days 1, 3, 5, and 7 in PBS (control)- and 2ccPA-treated mice. mRNA expression levels were normalised to those of *Gapdh* and were subsequently normalised to the corresponding expression levels of the contralateral hemisphere (n = 8 female mice/group). Each expression level in PBS-administered naïve mice was regarded as 1, and all other values were normalized relative to this value. *p < 0.05, **p < 0.01 vs. control group, two-way ANOVA and Tukey-Kramer test. Data represent the mean ± SEM of eight pairs of mice at each specified postoperative day. A total of 80 female mice (6 weeks old) were subjected to this experiment.

To understand the mechanisms underlying 2ccPA-induced attenuation of serum IgG extravasation in the injured cerebral cortex, an investigation was carried out to determine whether 2ccPA suppresses the expression of inflammatory cytokines in the vicinity of cortical stab wounds. It has been reported that mRNA expression levels of *Il-1β*, *Il-6*, *Tnf-α*, and *Tgf-β1* are increased in mouse cerebral cortices after application of a stab wound^20,26^. In the present study, mRNA expression levels of *Il-1β*, *Il-6*, *Tnf-α*, and *Tgf-β1* were quantified in TBI model mice receiving 2ccPA. Near the cortical lesions, the mRNA expression of the pro-inflammatory cytokines *Il-1β*, *Il-6*, and *Tnf-α* was increased in both control and 2ccPA-treated mice, reaching maximal values at day 1. However, the administration of 2ccPA significantly suppressed the mRNA expression of *Il-1β* on days 3–7, *Il-6* on days 5 and 7, and *Tnf-α* on day 5 (Fig. 2A–C). In addition, 2ccPA administration inhibited the injury-induced up-regulation of *Tgf-β1* mRNA expression on day 3 (Fig. 2D). In naïve mice, the cortical mRNA expression of *Il-1β*, *Il-6*, and *Tnf-α* was not significantly affected by the administration of 2ccPA at any investigated time point (Supplementary Fig. S2A–C).

### 2ccPA suppresses the activation of microglial cells after a stab wound injury

**Figure 3:**
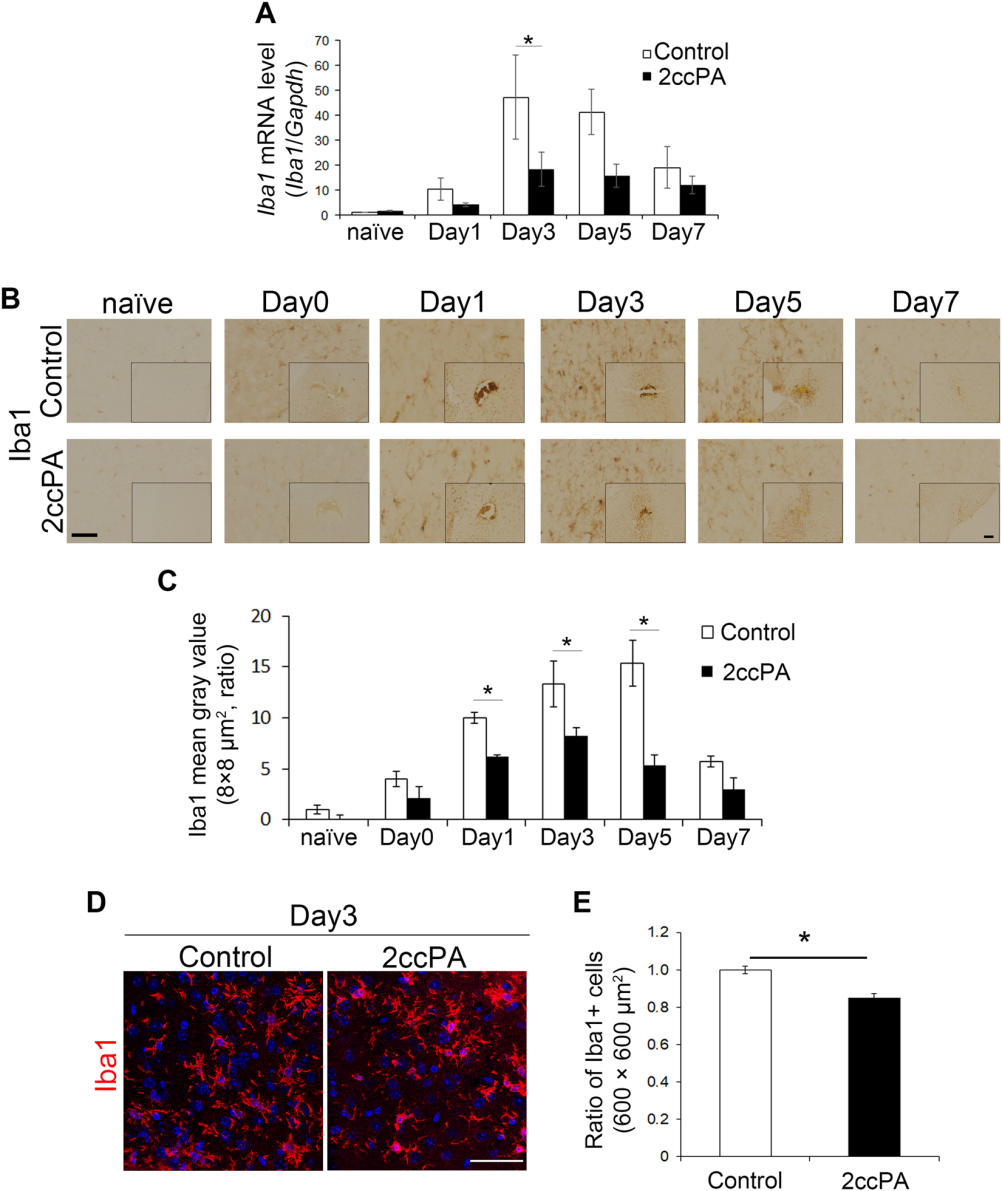
Effects of 2ccPA on Iba1 expression in microglial cells after a stab wound injury. (**A**) Real-time RT-PCR analysis of the *Iba1* mRNA levels near the injury site at days 1, 3, 5, and 7 in 2ccPA-treated mice. mRNA levels for *Iba1* were normalised to those for *Gapdh* and were subsequently normalised to the values in the corresponding contralateral region (n = 3 female mice/group). *p < 0.05, **p < 0.01 vs. control group, two-way ANOVA and Tukey-Kramer test. (**B**–**E**) Analysis of Iba1 expression near the stab wound site in mice receiving 2ccPA. (**B**) Cortical sections of PBS (control)- and 2ccPA-treated mice from postoperative days 0, 1, 3, 5, and 7 were immunoenzymatically stained with an antibody against Iba1. (**C**) Quantification of Iba1 staining intensities near the lesions. The mean grey values for Iba1 were normalised to those of the corresponding region in the contralateral hemisphere. After background normalisation, Iba1 mean grey values were normalised to those of control naïve mice (n = 3 female mice/group). *p < 0.05, **p < 0.01 vs. control group, two-way ANOVA and Tukey-Kramer test. (**D**) Representative examples of sections from PBS- and 2ccPA-treated mice on postoperative day 3 stained with anti-Iba1 antibodies shown in red. (**E**) Ratio of Iba1-positive cells to the total number of cells near stab wound regions on day 3 in PBS- and 2ccPA-treated mice. Scale bar: 100 µm. *p < 0.05, **p < 0.01 vs. control group, *t*-test. The RT-PCR data represent the mean ± SEM of eight pairs of mice at each specified day after the traumatic injury. All other data represent the mean ± SEM of three independent experiments using three pairs of mice at each postoperative day. A total of 110 female mice (6 weeks old) were subjected to this experiment.

Microglial cells are the major source of pro- and anti-inflammatory cytokines in the central nervous system^27,28^. It has been reported that the administration of cPA effectively attenuates the activation of microglial cells in the corpus callosum of mice in a multiple sclerosis model^14^. To determine whether 2ccPA also affects the activation of microglial cells in the cerebral cortex after a TBI, the effects of 2ccPA administration on cortical *Iba1* mRNA expression were examined. The administration of 2ccPA significantly suppressed the mRNA expression of *Iba1*, a microglial marker, on day 3 (Fig. 3A) but did not significantly affect the expression of *Iba1* mRNA in the cortices of naïve mice (Supplementary Fig. S2D). Next, the expression of Iba1 protein in the vicinity of stab wounds was examined using an immunoenzymatic staining technique. The administration of 2ccPA markedly suppressed the expression of Iba1 protein near stab wound regions on days 1, 3, and 5 (Fig. 3B,C). Furthermore, the number of Iba1-positive cells near injured regions was counted on day 3 using immunofluorescence-stained sections. The ratio of Iba1-positive cells to DAPI-positive cells was significantly decreased by 2ccPA (Fig. 3D,E).

### 2ccPA suppresses the expression of inflammatory cytokines in primary cultured microglial cells

**Figure 4:**
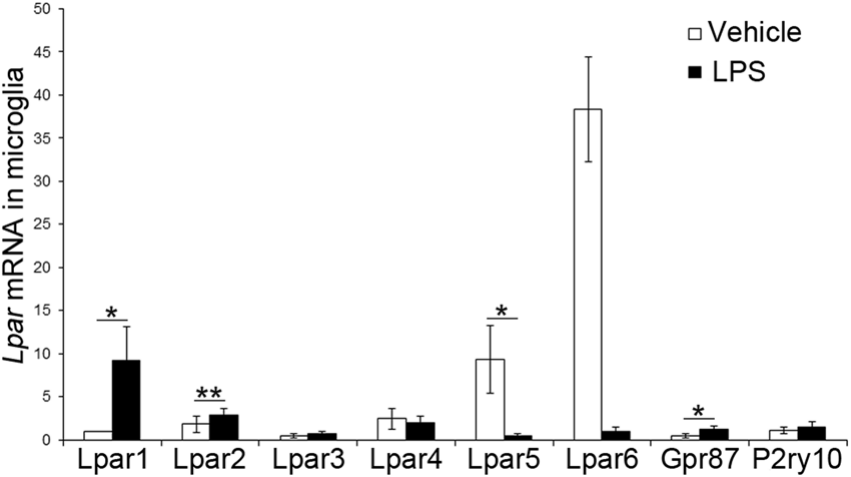
mRNA expression of putative 2ccPA receptors in primary cultured microglial cells. Real-time RT-PCR analysis of *Lpar1*, *Lpar2*, *Lpar3*, *Lpar4*, *Lpar5*, *Lpar6*, *Gpr87*, and *P2ry10* mRNA levels in cultured microglial cells exposed to LPS or PBS as a vehicle. Each mRNA expression level was normalised to that of *Gapdh*. All values are presented relative to the mRNA expression level of *Lpar1* in untreated microglial cells (control) (n = 3 cultures/group). *p < 0.05, **p < 0.01 vs. vehicle group, *t*-test. Data represent the mean ± SEM of three independent experiments. A total of 36 newborn mice (postnatal day 2) were subjected to this experiment.

**Figure 5:**
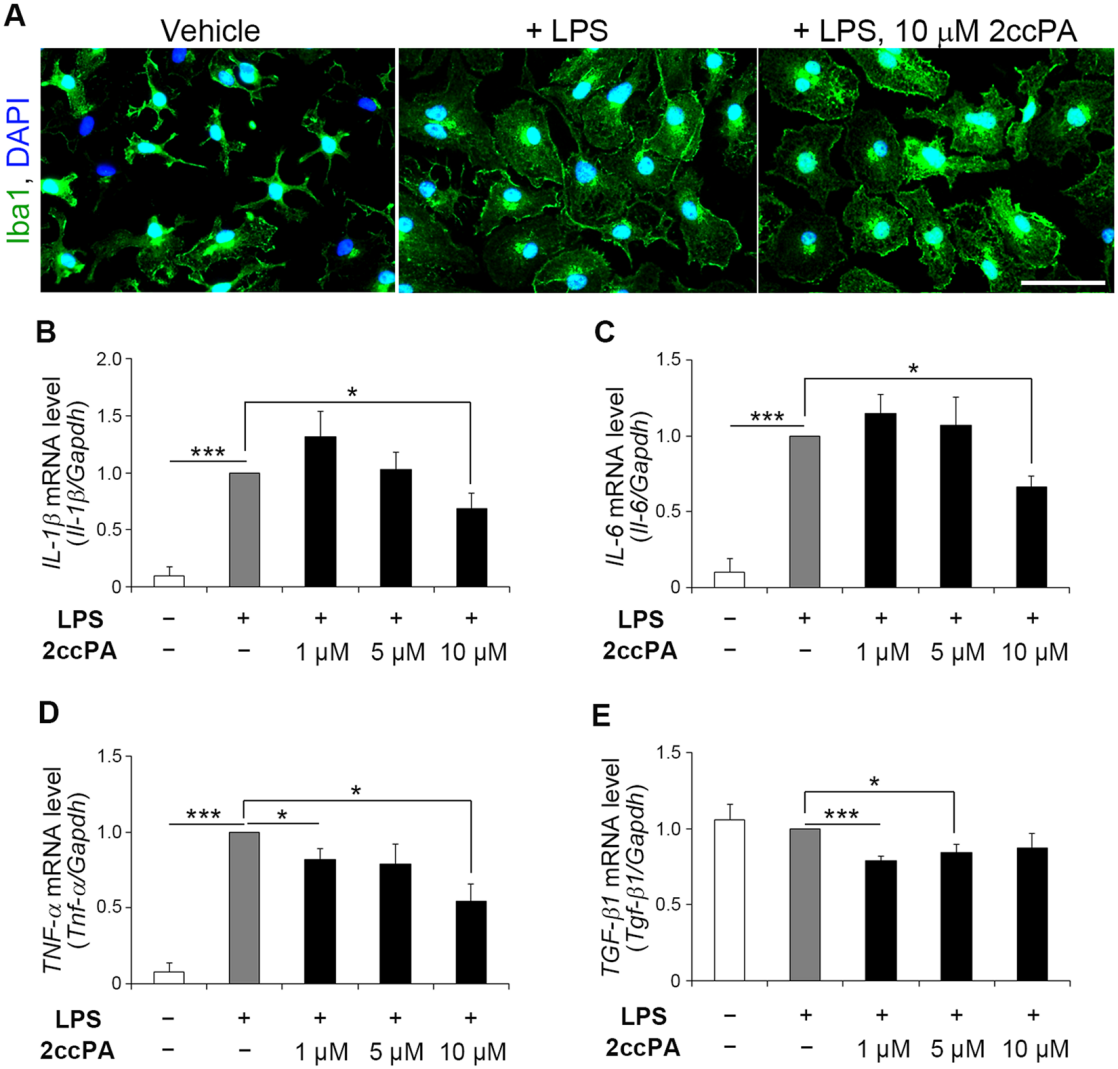
Effects of 2ccPA on mRNA expression of pro- and anti-inflammatory cytokines in primary cultured microglial cells. (**A**) LPS- and 2ccPA-stimulated microglial cells stained with anti-Iba1 antibody (green) and DAPI (blue). (**B,C,D,E**) Real-time RT-PCR analysis of *Il-1β*, *Il-6*, *Tnf-α*, and *Tgf-β1* mRNA expression in primary cultured microglial cells exposed to LPS and 2ccPA. The mRNA expression levels of *Il-1β*, *Il-6*, *Tnf-α*, and *Tgf-β1* were normalised to that of *Gapdh*. All mRNA levels were normalised to those from cultured microglial cells without LPS or 2ccPA (n = 3 cultures/group). Scale bar: 50 µm. *p < 0.05, **p < 0.01, ***p < 0.001 vs. control group, one-way ANOVA and Tukey-Kramer test. Data represent the mean ± SEM of five independent experiments. A total of 36 newborn mice (postnatal day 2) were subjected to this experiment.

To determine whether 2ccPA exerts its effects on the production of inflammatory cytokines via modulation of microglial cell behaviour, the effects of 2ccPA on the LPS-induced production of inflammatory cytokines in primary cultured microglial cells were examined. First, to confirm the expression of LPA receptors, the mRNA levels of the 2ccPA receptor candidates *Lpar1–6*, *Gpr87*, and *P*2*ry10* were measured in microglial cells with and without exposure to LPS. *Lpar1*, *Lpar2*, *Lpar3*, *Lpar4*, *Lpar5*, *Lpar6*, *Gpr87*, and *P2ry10* were expressed in cultured microglial cells in the presence and absence of LPS (Fig. 4), indicating that several candidates for 2ccPA receptors are expressed in microglial cells. The addition of LPS significantly increased the expression of *Lpar1*, *Lpar2* and *Gpr87* mRNA, and decreased *Lpar5* and *Lpar6* mRNA levels (Fig. 4). Next, the effect of 2ccPA on the mRNA expression of inflammatory cytokines was studied in cultured microglial cells. The addition of LPS changed the microglial cell morphology from ramified to amoeboid, the latter being considered a sign for microglial activation, and the presence of 10 μM 2ccPA did not appear to suppress this LPS-induced change (Fig. 5A). However, 10 μM 2ccPA suppressed the LPS-induced up-regulation of *Il-1β*, *Il-6*, and *Tnf-α* mRNA expression in microglial cells (Fig. 5B–D). The addition of 5 μM 2ccPA to LPS-treated microglial cells did not affect the *Il-1β*, *Il-6*, *Tnf-α* mRNA levels (Fig. 5B–D). Moreover, the expression of *Tgf-β1* mRNA was also decreased by the addition of 1 and 5 μM 2ccPA (Fig. 5E).

### 2ccPA promotes the progression from the M1 to the M2 phenotype in microglial cells after TBI

**Figure 6:**
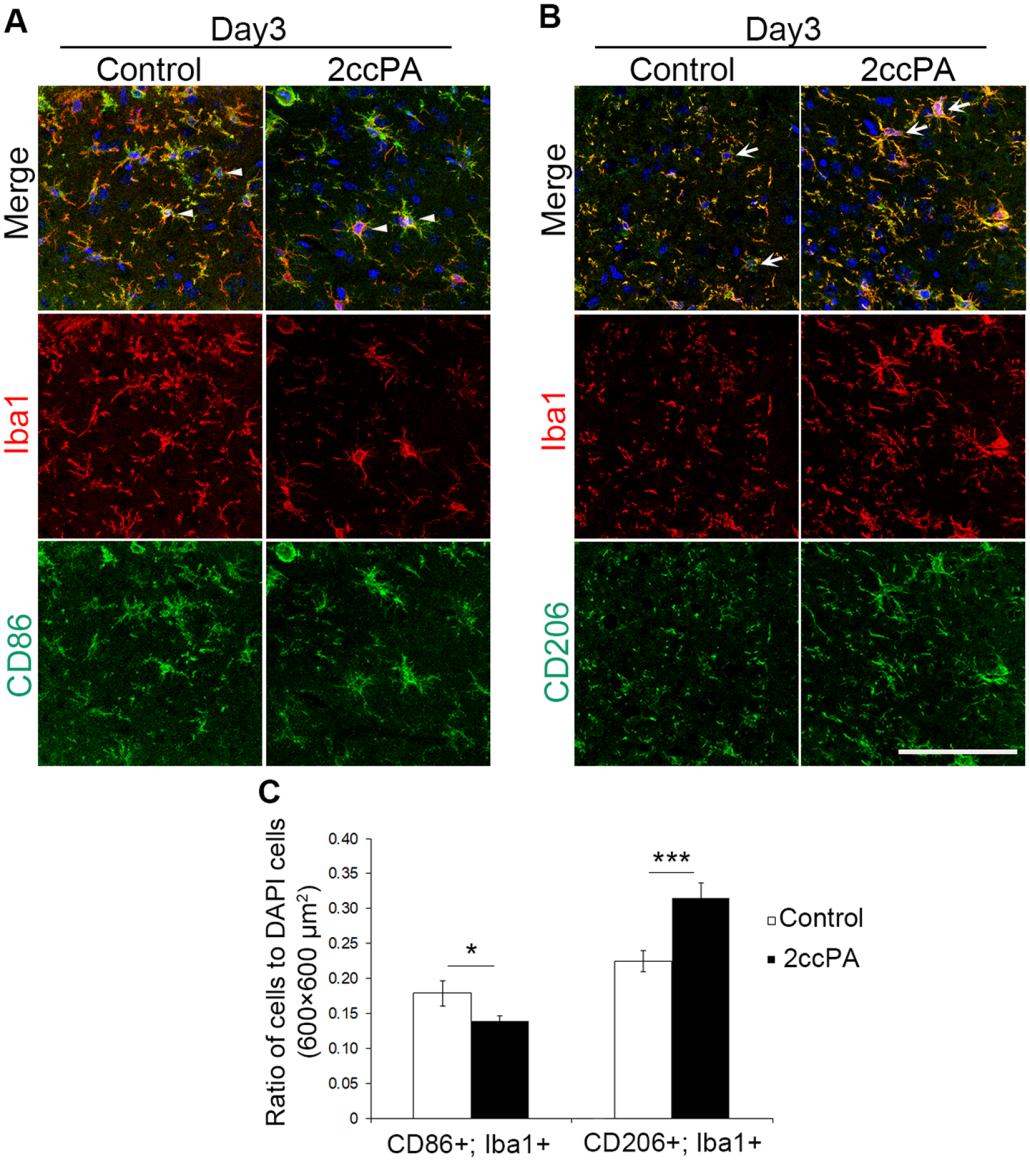
Effects of 2ccPA on microglial polarisation towards M1 or M2 phenotypes after a stab wound injury. (**A**) Immunofluorescent images of cerebral cortices from PBS (control)- and 2ccPA-treated mice on postoperative day 3. Sections were stained with anti-CD86 (green), anti-Iba1 (red), and DAPI (blue). (**B**) Immunofluorescence staining of cerebral cortex sections from PBS- and 2ccPA-treated mice on day 3 with anti-CD206 (green), anti-Iba1 (red), and DAPI (blue). The arrowheads and arrows indicate CD86+/Iba1+ and CD206+/Iba1+ cells, respectively. (**C**) Ratios of CD86+/Iba1+ or CD206+/Iba1+ cell numbers to DAPI+ cell numbers near the stab wounded injury on day 3 in PBS- and 2ccPA-treated mice (n = 3 female mice/group). Scale bar: 100 µm. *p < 0.05, ***p < 0.001 vs. control group, *t*-test. Data represent the mean ± SEM of three independent experiments using three pairs of mice. A total of 6 female mice (6 weeks old) were subjected to this experiment.

Activated microglial cells can be categorised into two opposite types, an M1 phenotype, which promotes inflammation, and an M2 phenotype, which produces anti-inflammatory cytokines and protects neurons^29–31^. To determine whether 2ccPA modulates microglial polarisation in the vicinity of a stab wound towards the M1 or M2 phenotype, the numbers of CD86− and CD206−positive cells, which are the markers of M1 and M2 phenotype, respectively, were quantified near the lesion site on day 3. The ratio of CD86+/Iba1+ cells to DAPI+ cells near the site of the injury was significantly decreased by administration of 2ccPA. Additionally, the ratio of CD206+/Iba1+ cells to DAPI+ cells was significantly increased by 2ccPA administration (Fig. 6).

## Discussion

We studied the effects of 2ccPA administration on wound healing in the murine cerebral cortex after a stab wound injury using as parameters the degree of haemorrhage, inflammation, and activation of microglial cells. First, we observed that the administration of 2ccPA significantly attenuates the extent of BBB breakdown caused by a stab wound (Fig. 1), suggesting that 2ccPA promotes recovery from an injury-induced breakdown of the BBB. Second, 2ccPA suppresses the TBI-induced up-regulation of mRNA for inflammatory cytokines such as *Il-1β*, *Il-6*, *Tnf-α*, and *Tgf-β1* (Fig. 2), suggesting that 2ccPA attenuates the inflammation after a stab wound injury. Third, we determined that the administration of 2ccPA markedly attenuates the injury-induced up-regulation of the microglial cell number and the Iba1 protein level (Fig. 3B–E). In our study, 2ccPA was not only administered shortly after the induction of the traumatic injury but also repeatedly at intervals of 24 h until the collection of the brains. Therefore, a continuous administration of 2ccPA might be needed to maintain its effects during wound healing. Fourth, 10 μM 2ccPA suppresses the LPS-induced up-regulation of mRNA levels from inflammatory cytokines in cultured microglial cells (Fig. 5). This suggests that 2ccPA attenuates the inflammation *via* regulation of microglial activation in the cerebral cortex after a traumatic injury. Fifth, the administration of 2ccPA in injured mice decreases the number of M1 microglial cells, while increasing simultaneously the number of microglial cells with an M2 phenotype (Fig. 6), suggesting that 2ccPA modulates microglial polarisation towards M1 or M2 phenotypes. These data suggest that 2ccPA administration leads to a suppression of BBB breakdown extent and inflammation caused by a stab wound injury.

Regarding the observed 2ccPA-induced suppression of BBB breakdown in the stab wound region, it has been suggested that 2ccPA accelerates the recovery from BBB breakdown. However, the underlying mechanisms are unknown. The extracellular matrix is one of the basic components of the BBB and plays a critical role in maintaining its integrity. Several studies have reported that proteolytic enzymes, such as matrix metalloproteinases (MMPs) and urokinase plasminogen activator (uPA), are up-regulated after brain injury and degrade the BBB^32^. Studies have shown that LPA increases MMP-9 and uPA expression and thus increases the permeability of BBB^32^. Furthermore, it has been reported that LPA levels are not only elevated in a mouse model of TBI, but also in the cerebrospinal fluid of TBI patients^2^. Since cPA is an endogenous inhibitor of LPA production through ATX^15^, 2ccPA might suppress LPA activity and thereby offer protection against BBB breakdown.

We found that 2ccPA suppresses not only the severity of BBB breakdown but also the inflammation in the vicinity of the stab wound injury. In the present study, it was determined that the mRNA expression of the pro-inflammatory cytokines *Il-1β*, *Il-6*, and *Tnf-α* were suppressed by 2ccPA administration in TBI model mice (Fig. 2). We also found that the activation of microglial cells induced by the stab wound was attenuated by 2ccPA (Fig. 3). These findings suggest that 2ccPA prevents the up-regulation of pro-inflammatory cytokine expression *via* a suppression of microglial cell activation. This is supported by the fact that 2ccPA also suppresses the mRNA expression of pro-inflammatory cytokines in primary cultures of LPS-stimulated microglial cells (Fig. 5B–D). Since pro-inflammatory cytokines are also produced by astrocytes^27,28^, it is possible that the administration of 2ccPA suppresses the activation of astrocytes. Further experiments are required to determine the effects of 2ccPA on the activation of astrocytes after a stab wound injury.

It is possible that 2ccPA plays a role in the modulation of microglial polarisation towards the M1 or M2 phenotype. Our results suggest that 2ccPA modulates microglial polarisation towards the M2 phenotype rather than M1 phenotype. It is known that 2ccPA and cPA protect oligodendrocytes and neuroblastoma Neuro2A cells *via* suppression of apoptosis pathways^12,17,33^, suggesting that 2ccPA exerts a neuroprotective activity. Since M2 microglial cells play roles in attenuating inflammation and promoting tissue repair^34^, our data suggest that 2ccPA contributes to the suppression of inflammation after the stab wound injury *via* promotion of microglial polarisation towards the M2 phenotype in the mouse cerebral cortex. It is known that M2 microglial cells release anti-inflammatory cytokines such as TGF-β1^35^. In the present study, 2ccPA did not increase *Tgf-β1* mRNA expression *in vivo* or *in vitro* (Figs 2D, 5E) despite the observed increase in the number of M2 microglial cells. In control mice, the injury-induced increase of mRNA levels from pro-inflammatory cytokines peaked on day 1. While the pro-inflammatory mRNA expression began to decrease on day 3, the mRNA level of the anti-inflammatory cytokine TGF-β1 reached its maximum values (Fig. 2), suggesting that TGF-β1 expression is up-regulated by inflammation in regions near the stab wound. This is in support of the notion that the lack of TGF-β1 up-regulation in TBI model mice might be caused by a 2ccPA-induced attenuation of the inflammation.

We found that mouse cerebral cortices and microglial cells express receptors for 2ccPA including LPA1–6, GPR87, and P2Y10, which are known to be activated by cPA^15,36–39^. LPA receptors mRNA expression levels were increased until day 5 after the stab wound trauma and declined to basal levels at day 7 after the injury (Supplementary Fig. S1). These increases in mRNA levels of LPA receptors were suppressed by 2ccPA treatment (Supplementary Fig. S1). In this study, we were not able to establish, whether 2ccPA administration decreases the mRNA expression of LPA receptors directly or indirectly by suppression of the inflammation. To our knowledge, there is no report that 2ccPA and cPA regulate the expression of LPA receptors. Therefore, it might be necessary to investigate in future studies whether 2ccPA controls the expression of LPA receptors directly. Furthermore, the addition of LPS altered the expression of these receptors for 2ccPA (Fig. 4). Our data indicate that LPS increases *Lpar1*, *Lpar2*, and *Gpr87* mRNA expression levels in cultured microglial cells. The selective blockade of LPA1 results in reduced demyelination and improvement in locomotor recovery after spinal cord contusion in mice^40^. It is possible that 2ccPA binds to LPA1, LPA2, GPR87 in LPS-stimulated microglial cells and thereby suppresses inflammation.

The mechanisms underlying 2ccPA suppression of pro-inflammatory cytokine production are unclear. LPS binds to toll-like receptor 4 (TLR4) and promotes the transcription of pro-inflammatory cytokines *via* nuclear factor-kappa B (NF-κB) and activator protein 1 (AP-1) pathways^41,42^. The transcription of inflammatory cytokines might be suppressed by 2ccPA *via* inhibition of the activation of signal transduction factors in the NF-”κB and AP-1 pathways. In addition, it might be an important topic for future studies to elucidate the mechanisms by which 2ccPA regulates the microglial polarisation towards an M1 or M2 phenotype. NF-κB regulates the expression of M1-related genes^43^. Knockout of TLR4 decreases the phosphorylation of NF-κB and increases the number of M2 type microglial cells, indicating that the activity of NF-κB is involved in microglial polarisation^44^. Therefore, it is possible that 2ccPA decreases the polarisation into M1 type microglia by the inhibition of NF-κB activity. Moreover, it is known that signal transducers and activators of transcription 3 (STAT3) regulates the microglial polarisation into the M1 type, while signal transducers and activators of transcription 6 (STAT6) promotes microglial M2 polarisation^43^. It might be of interest to study the influence of 2ccPA on the nuclear translocation of the STAT family during M1 and M2 polarisation of microglial cells.

In summary, we examined the effects of 2ccPA on the wound healing of the cerebral cortex in a mouse model of TBI. This study indicates that administration of 2ccPA significantly attenuates the severity of BBB breakdown, suppresses the up-regulation of mRNA expression from pro-inflammatory cytokines, attenuates the number of neurotoxic M1 microglial cells, and increases the number of neuroprotective M2 microglial cells. These findings demonstrate that 2ccPA suppresses primary and secondary traumatic brain injury *via* regulation of microglial activation in the cerebral cortex.

## Electronic supplementary material

Supplementary information
