# Supplementary material for: 2-carba cyclic phosphatidic acid suppresses inflammation via regulation of microglial polarisation in the stab-wounded mouse cerebral cortex
Source: Sci Rep. 2018 Jun 26;8:9715. doi: 10.1038/s41598-018-27990-1 (PMC6018705; doi:10.1038/s41598-018-27990-1)
Supplement: Supplementary file 1 — Supplementary information [file 41598_2018_27990_MOESM1_ESM.docx]

SUPPLEMENTARY INFORMATION

**2-carba cyclic phosphatidic acid suppresses inflammation *via* regulation of microglial polarization in the stab-wounded mouse cerebral cortex**

Kei Hashimoto, Mari Nakashima, Ayana Hamano, Mari Gotoh, Hiroko Ikeshima-Kataoka, Kimiko Murakami-Murofushi, Yasunori Miyamoto


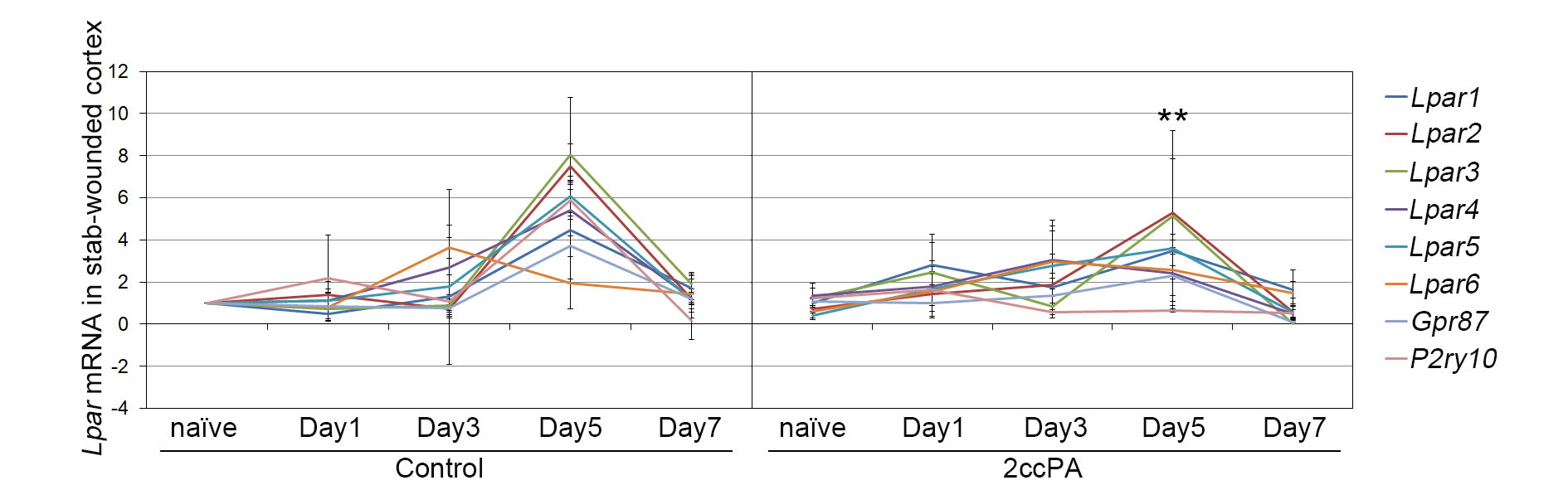


**Figure. S1.** The mRNA expression of the putative 2ccPA receptors, *Lpar1-6*, *Gpr87*, and *P2ry10* in mouse cerebral cortices after a stab wound injury. Real-time RT-PCR analysis of *Lpar1-6*, *Gpr87*, and *P2ry10* mRNA expression levels in the cerebral cortices of PBS (control)- and 2ccPA-treated mice at postoperative days 1, 3, 5, and 7 (n = 3 female mice/group). The *Lpar1-6*, *Gpr87*, and *P2ry10* mRNA expression levels were normalised to that of *Gapdh* and were subsequently normalised to the *Lpar1-6*, *Gpr87*, and *P2ry10* expression levels in the corresponding region of the contralateral hemisphere. All values were normalised to the corresponding expression level in PBS-treated naïve mice. **: *p* < 0.01 vs. control group, two-way ANOVA. Data represent the mean ± SEM of three pairs of mice at each specified day after the stab wound injury. A total of 30 female mice (6 weeks old) were subjected to this experiment.


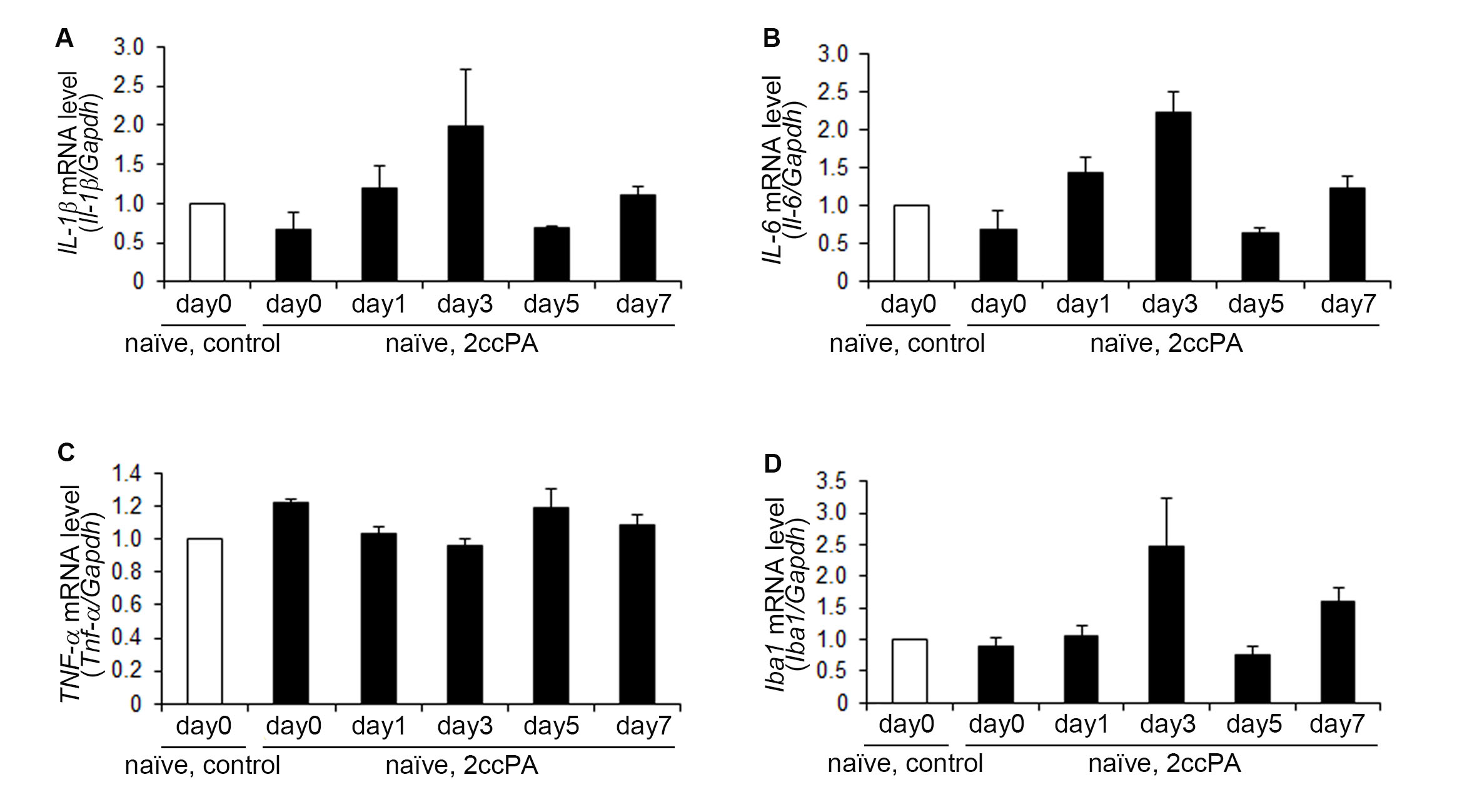


**Figure. S2.** The mRNA expression of pro-inflammatory cytokines and *Iba1* in the cerebral cortices of 2ccPA-treated naïve mice. (A-D) Real-time RT-PCR analysis of *Il-1β*, *Il-6*, *Tnf-α*, and *Iba1* mRNA expression in the cerebral cortices of PBS (control)- and 2ccPA-treated mice. The brains of control mice were collected immediately after PBS administration on day 0. The successive administration of 2ccPA was performed at intervals of 24 h, and the brains were collected on days 0, 1, 3, 5, and 7. The *Il-1β*, *Il-6*, *Tnf-α*, and *Iba1* mRNA expression levels were normalised to that of *Gapdh* and were subsequently normalised to the *Il-1β*, *Il-6*, *Tnf-α*, and *Iba1* expression levels in the contralateral hemisphere (n = 2 female mice/group). All values were normalised to the expression levels in naïve control mice. Data represent the mean ± SEM of two pairs of mice at each specified day after the stab wound injury. A total of 12 female mice (6 weeks old) were subjected to this experiment.
